# A Path to Resilience: The Impact of School-Based Yoga on Adolescent Mental Well-Being

**DOI:** 10.7759/cureus.73604

**Published:** 2024-11-13

**Authors:** Selvaraj Giridharan, Bhuvana Pandiyan

**Affiliations:** 1 Oncology, Tawam Hospital, Al Ain, ARE; 2 Psychiatry, Herefordshire and Worcestershire Health and Care NHS Trust, Hereford, GBR

**Keywords:** adolescents, emotional regulation, mental health, resilience, stress reduction, yoga

## Abstract

Adolescent mental health is an increasing global concern, with rising levels of stress, anxiety, and depression driven by academic pressure and social expectations. School-based yoga programs are accessible and efficacious interventions for improving mental well-being. Systematic reviews and randomised controlled trials have indicated that yoga interventions improve psychological, cognitive, and physical outcomes in youth, addressing both preventive and therapeutic mental health needs. However, given the diversity of yoga styles and protocols, further research is required to identify the specific types or components of yoga that are most effective and feasible in school-based programs. These benefits stem from yoga's impact on stress regulation, emotional awareness, social interactions, and physical well-being. Practical considerations for school implementation include age-appropriate design, teacher training, inclusivity, and parental involvement. With substantial evidence supporting the role of yoga in adolescent mental health, schools can integrate it into their wellness curriculum. As mental health issues persist, school-based yoga offers a proactive approach to fostering resilience and assisting youths in navigating life challenges. This editorial reviews evidence on the benefits of yoga for adolescents, emphasising emotional regulation, stress reduction, and resilience.

## Editorial

Introduction

Adolescent mental health is a significant global concern, with increasing stress, anxiety, and depression due to academic pressure, social expectations, and the COVID-19 pandemic. Adolescents who are vulnerable to emotional and behavioural issues often engage in high-risk behaviours during identity development. Mental health problems account for approximately 7.5% of the global disease burden of children. Adolescent stress and anxiety are increasing in populous countries, such as India and China, with higher reports of self-harm and mental health issues. Surveys in India revealed high anxiety levels among schoolchildren, while in China, adolescent depression rates reached up to 24% in some regions. In England, 16% of children aged 5-16 have been diagnosed with mental health disorders as of 2020 [1,2]. This increasing mental health burden among adolescents highlights the need for accessible and efficacious interventions in the educational system. Yoga, an ancient practice encompassing physical postures, breathwork, and mindfulness, has gained attention for its holistic benefits to mental and physical well-being. It is increasingly regarded as an effective intervention for fostering resilience, emotional regulation, and stress management in adolescents [3,4]. Research elucidates yoga's role not only in addressing physical health but also in supporting cognitive and emotional regulation competencies particularly salient for adolescents navigating developmental transitions and the complexities of contemporary life [1,5].

School-based yoga programs have demonstrated the potential to support adolescent mental health by offering an accessible form of intervention within an educational environment. Evidence from systematic reviews indicates that yoga interventions can reduce stress, improve mood, and enhance sleep and relaxation among students [3,4]. These programs provide both preventive and therapeutic support for adolescent well-being, emphasising yoga's potential as a holistic and inclusive approach to mental health in educational settings [5].

Evidence of effectiveness

Extensive evidence demonstrates the effectiveness of yoga in reducing stress, improving emotional regulation, and enhancing adolescent well-being. Research has shown that yoga aids in anger management and boosts self-confidence among adolescents. Practices such as mindful breathing and guided relaxation improve anger control and help adolescents develop adaptive responses to emotional triggers. Yoga's focus on self-awareness and physical achievement also enhances self-esteem and confidence, acting as protective factors against risk-taking behaviours. A systematic review by Khunti et al. of 21 school-based yoga intervention studies found significant improvements in mental health indicators such as stress, anxiety, and low mood in children and adolescents [2]. Similarly, Miller et al. conducted a review of 39 RCTs, which demonstrated that yoga improved psychological, cognitive, and physical functioning outcomes in youth, further establishing yoga as a promising intervention for mental health [5]. Research has also elucidated the physiological impact of yoga on stress regulation. For instance, a study by Hagen et al. on the effects of school-based yoga revealed that adolescents reported increased mindfulness and awareness of their need to relax, which led to improvements in relaxation and sleep quality [4]. This focus on relaxation aligns with the salutogenic model, which emphasises the enhancing factors that promote health rather than solely treating symptoms. Additionally, qualitative studies have suggested that yoga promotes interpersonal skills, empathy, and positive social interactions, which are essential for adolescent development. Patra et al. highlighted that adolescents in yoga programs exhibited greater emotional awareness and empathy and improved social communication, fostering positive social dynamics within school environments [1]. Despite methodological limitations, such as small sample sizes and intervention variability, the existing evidence consistently indicates yoga's role in supporting mental health in an accessible, non-stigmatising manner.

Mechanisms of yoga's impact 

The efficacy of yoga in promoting adolescent mental health is postulated to stem from its combined effects on both mind and body, which addresses the distinctive stressors encountered by this age group. The therapeutic potential of yoga is derived from its holistic approach, which incorporates physical posture, respiratory techniques, and mindfulness practices.

Physiological Modulation of the Stress Response

Yoga's stress-reducing properties are primarily attributed to its modulatory effect on the autonomic nervous system. By harmonising sympathetic and parasympathetic responses, yoga facilitates a reduction in cortisol, the principal stress hormone. This attenuation of cortisol levels aids adolescents in mitigating the chronic stress associated with scholastic demands and social complexities, thereby enhancing their psychological resilience [2,3].

Augmentation of Emotional Regulation and Mindful Awareness

Yoga cultivates mindfulness, facilitating heightened awareness of cognitive and affective states among adolescents. Enhanced self-awareness can promote the management of impulsivity and foster the development of adaptive coping strategies. Structured breathing exercises and meditative elements inherent in yoga practice serve as instrumental tools for emotional regulation, anxiety reduction, and enhancement of mental fortitude [5].

Enhancement of Social Competence and Interpersonal Dynamics

The collective nature of yoga practice engenders social interactions and empathic understanding. This aspect of yoga can cultivate a sense of belonging and social support, which is paramount for adolescents navigating complex social landscapes. Empirical evidence suggests that adolescents engaged in yoga programs exhibit increased empathy and enhanced social communication skills, which are crucial components of their social development [1,4].

Promotion of Physical Health and Body Image

As a form of physical exercise, yoga enhances body awareness, flexibility, and muscular strength. These somatic benefits are intricately linked to self-esteem and body image, which are particularly salient, given the prevalence of body-related concerns among adolescents. Positive alterations in physical health can engender a more favourable self-perception and corporeal relationship, thereby contributing to enhanced psychological well-being [5].

Collectively, these mechanisms elucidate yoga's comprehensive approach to adolescent mental health, addressing both the somatic and psychological domains. This multifaceted support equips adolescents with the requisite tools for stress management, emotional regulation, and the cultivation of resilience.

Opportunities and challenges

The integration of yoga programs into educational institutions presents a promising avenue for supporting adolescent mental health in structured, familiar environments. However, maintaining consistent student participation or adherence to such programs can be challenging. 

Designing Age-Appropriate and Engaging Programs

Adolescents may experience diminished interest over time or feel self-conscious about participating in yoga during school hours. To address this, educational institutions should focus on developing engaging, age-appropriate, and diverse yoga sessions. Additionally, incorporating brief, regular sessions into the school schedule as part of physical education or wellness classes can help normalise participation [2]. Fostering a supportive environment in which yoga is practised collectively may also improve adherence by cultivating a sense of community and mitigating any stigma associated with participation.

Teacher Training and Support

Effective implementation necessitates trained instructors to safely guide students through yoga practices. Educational institutions should consider training school counsellors or mental health professionals to facilitate yoga and integrate it with other mental health resources [4].

Addressing Accessibility and Inclusivity

Yoga is a cost-effective practice requiring minimal equipment, rendering it accessible to schools with varying resources [4]. Offering yoga as an optional component of physical education or wellness courses ensures that students from diverse backgrounds and fitness levels can participate.

Evaluating Program Impact and Sustainability

To ensure sustainability, educational institutions should evaluate yoga's impact on mental health. Collaboration with academic institutions can facilitate the assessment of program outcomes and contribute to the evidence-based development of school-based yoga interventions [1].

Engaging Parents and Addressing Cultural Perceptions

Some parents may perceive yoga as a cultural or religious practice. Transparent communication regarding the health-focused and evidence-based benefits of yoga can foster parental support. Information sessions or newsletters elucidating the program’s objectives may help address these concerns [5].

Future directions 

Future development of school-based yoga programs should encompass longitudinal studies to assess their long-term impact on mental health, resilience, and academic outcomes, thereby providing insights into how sustained practice benefits adolescents. To enhance efficacy, tailored interventions that combine various aspects of yoga, such as physical postures, breathwork, and relaxation techniques, alongside awareness and mindfulness sessions, may provide a more comprehensive approach to mental well-being. Such customised programs could be particularly beneficial for addressing specific needs such as anxiety or trauma. Additionally, incorporating digital platforms may increase accessibility to remote or underserved students, allowing for a more flexible and inclusive approach to yoga-based mental health support.

Conclusion

Evidence supports yoga as an effective intervention for adolescent mental health, aiding stress management, emotional regulation, and overall well-being. With rising mental health issues among youth, schools are well placed to implement preventive and supportive measures such as yoga. Schools and policymakers should integrate yoga into wellness curricula, support efficacy research, and promote inclusive culturally sensitive practices. Yoga enhances resilience and self-awareness, provides tools for life challenges, and promotes healthier development. Future research should conduct longitudinal studies to explore the long-term impact of school-based yoga on adolescent development, resilience, and mental well-being.
